# Feasibility and Effectiveness of the Web-Based WeActive and WeMindful Interventions on Physical Activity and Psychological Well-Being

**DOI:** 10.1155/2021/8400241

**Published:** 2021-10-04

**Authors:** Michele W. Marenus, Andy Murray, Kathryn Friedman, Julia Sanowski, Haley Ottensoser, Ana Cahuas, Varun Kumaravel, Weiyun Chen

**Affiliations:** ^1^School of Kinesiology, University of Michigan, Ann Arbor, MI 48109, USA; ^2^Department of Psychology, University of Michigan, Ann Arbor, MI 48109, USA

## Abstract

This study is aimed at examining the feasibility and effectiveness of aerobic and resistance training (WeActive) and mindful exercise (WeMindful) interventions in improving physical activity (PA), psychological well-being (PWB), and subjective vitality among college students. Participants in this study were 77 college students who were randomly assigned to either the WeActive group (*n* = 43) or the WeMindful group (*n* = 28). The WeActive group attended two 30-minute aerobic and resistance training sessions per week, and the WeMindful group attended two 30-minute yoga and mindful exercise sessions per week for eight weeks. All participants completed the *International Physical Activity Questionnaire*, the *World Health Organization-Five Well-Being Index*, and the *Subjective Vitality Scale* before and after the intervention, as well as the *Assessing Feasibility and Acceptability Questionnaire* at the end of the intervention. The primary study outcome measures were PA, PWB, and subjective vitality. A repeated-measures ANCOVA indicated a significant main effect of time for total PA (*F* = 7.89, *p* = 0.006, *η*^2^ = 0.049), vigorous PA (*F* = 5.36, *p* = 0.024, *η*^2^ = 0.022), and walking (*F* = 7.34, *p* = 0.009, *η*^2^ = 0.042) in both intervention groups. There was a significant interaction effect of time and group for PWB (*F* = 11.26, *p* = 0.001, *η*^2^ = 0.022), where the WeActive group experienced a decrease in PWB scores while participants in the WeMindful group experienced an increase in PWB scores over time. There was a main effect of group for subjective vitality (*F* = 8.91, *p* = 0.007, *η*^2^ = 0.088), indicating that the WeMindful group experienced a greater increase in subjective vitality than the WeActive group. Further, the participants in both groups indicated that the synchronized and asynchronized Zoom-based WeActive and WeMindful interventions were acceptable, appropriate, and feasible for participants. This study demonstrated that mindful exercise is effective in increasing PA, PWB, and subjective vitality while aerobic and resistance training may only be effective in increasing PA.

## 1. Introduction

Psychological health treatment and prevention have been the forefront of research and university administrations' focus due to the growing rates of mental illness observed in college students [1–3]. College students face unique stressors during this transitory period and often set habits that last beyond their college years [4, 5]. Most research on psychological health has focused on the presence and treatment of mental illness and has framed well-being as simply the absence of negative functioning [6]. Many researchers argue that psychological health should not just be limited to the lack of dysfunction—it should also focus on the positive aspects of mental health such as psychological well-being (PWB) [7–9]. PWB can be broadly defined as the “optimal psychological functioning and experience” (p. 142), which includes dimensions such as life satisfaction and personal growth [9, 10]. PWB is characterized by two distinct theoretical perspectives: the eudaimonic approach and the hedonic approach [9]. The eudaimonic approach defines well-being according to the ability of one to identify the life pursuits that are meaningful to them and strive to achieve their best. Vitality, the state of feeling alive and alert, is highly related to eudaimonic well-being [11]. The hedonic approach defines well-being according to pleasure and happiness being pursued and attained. Factors that contribute to one's PWB will differ throughout different ages and different circumstances, making approaches to improving PWB unique to each population [12].

Physical activity (PA) has shown to be an effective way to improve PWB as PA can improve mental health issues such as anxiety and depression and increases well-being [12, 13]. A study on 925 college students in China found that participants' hope, gratitude, life satisfaction, and happiness were significantly related to healthy body weight [14]. An additional study on college students demonstrated that PA was positively associated with higher levels of life satisfaction and happiness. Physical health—which can be maintained through PA—was found to be a positive predictor of PWB in college students [15]. Further, studies have shown that moderate to vigorous PA provides the greatest physical and mental health benefits [16–18]. During the transition to adulthood, research has shown a substantial decrease in PA for young adults [19]. This behavioral change is of particular importance because PA is a modifiable risk factor for poor PWB [20]. Throughout the recent decades, there has been a worldwide increase in obesity, a warning signal of blood pressure diseases, arthritis, cancer, type II diabetes, and CVDs [21], and recent studies have warned about the reduction of physical activities among college students [22]. Therefore, helping university students maintain higher PA from adolescence into adulthood is a major public health challenge.

The spread of the COVID-19 pandemic caused an unprecedented impact on daily life, presenting significant challenges to the development and maintenance of healthy behaviors, especially in college students [23]. COVID-19 has uniquely impacted college students due to insecurity and uncertainty around housing, academics, and future career options [23, 24]. Higher rates of mental illness and distress are typically observed in college students even prior to COVID-19, making them a vulnerable population to the detrimental effects of the pandemic [24, 25]. A systematic review reported that five studies showed a reduction of between 32% and 365.5% in light/moderate walking PA and seven studies showed a reduction between 2.9% and 52.8% reduction in high/vigorous PA, when compared to the prelockdown levels in university students [26]. Due to the COVID-19 pandemic confinements for university students, walking and moderate, vigorous, and total PA levels have been reduced [26]. One study found that individuals who reported over 30 minutes per day of moderate to vigorous PA were less likely to have symptoms of anxiety or depression [27]. Further, there was an observed decrease in PA in Italy, resulting in a negative effect on the well-being of the population [28]. Studies assessing the effects of the pandemic on college students are calling for the development of interventions and preventative measures to address physical and mental health concerns [29].

Different types of exercise have been found to be beneficial for mental and physical health. Due to the stigma associated with a diagnosis of a mental illness, many students may avoid obtaining help for their poor PWB, anxiety, and depression [30]. Mind-body practices, such as yoga, have been shown to help college students cope with stress, anxiety, and depression, which are all related to PWB [31]. Mindful exercises often incorporate mindfulness. Mindfulness is defined as “the awareness that emerges through paying attention on purpose, in the present moment, and nonjudgmentally to the unfolding of experience moment by moment” [32] (p. 145). Mindfulness practices have been shown to increase mindful awareness, reduce psychological distress, and improve PWB and subjective vitality in college students [33]. Mindful exercises have been recommended as a nonpharmaceutical practice that can support student well-being [34]. Research examining the relationships between yoga practice and well-being found that there was a correlation between the number of years practicing yoga and gratitude and meaning in life [35]. Further, an intervention conducted with 20 healthy college students found that participants who attended a 90-minute yoga class twice per week for eight weeks experienced significant improvements in their positive and negative effects [36]. Another study on college students in India found that participants saw significant improvement in PWB after completion of six 45-minute yoga sessions each week for three months, compared to a control group [37]. This research shows the overall positive impact of mind-body exercises.

Studies have also shown that aerobic and resistance training exercises have positive effects on the mental health and well-being of college students [38–40]. In a randomized control trial study of effects of a six-week aerobic exercise program in 30 female college students, the experimental group (*n* = 15) had greater improvement in mental health indices such as social function, anxiety, and depression compared to the control group (*n* = 15) [22]. A study on 44 women, who were assigned to either aerobic dance exercise class three times per week for eight weeks or a control group, found that the participants in the experimental group showed significant improvements in their eudaimonic well-being, compared to the control counterparts [41]. Similarly, other studies have observed psychological benefits after participating in resistance training in adults [42]. One intervention study that assessed the impact of 12-week resistance training sessions found that participants who attended at least twice per week saw slight improvements in mental health, compared to the control group [43]. In short, studies have indicated that both aerobic exercise and resistance training may enhance overall well-being. Given the limited number of intervention studies that examine the effects of aerobic and resistance training on PWB in college students, there is a need to further research in this area.

Web-based physical activity interventions may be particularly effective in promoting health behaviors in college students [39, 44]. A systematic review found that providing virtual PA programs improves limitations that are traditionally experienced during in-person or print-based interventions while maintaining similar effectiveness outcomes. Some of these improvements include increased flexibility and the ability to access more people at a low cost [45]. A study on college students in Hong Kong supported the feasibility of internet-based interventions in promoting mental health [46]. They found that an 8-week 30-to-45-minute internet-based mindfulness training significantly enhanced mental well-being at posttest and 3-month follow-up when compared with the waitlist control [46]. Additionally, reminder emails have been found to enhance the effectiveness of the intervention if they are proactively sent to participants during the duration of the web-based intervention [47]. A meta-analysis of e-health interventions found that internet-based PA interventions are effective in increasing PA participation in young people [48]. Of the 10 studies included in the meta-analysis, six studies were randomized control trials, three were quasiexperimental, and one was a cluster randomized control. Eight of the 10 studies reported increases in PA and showed sustainment up to eight months postintervention [48]. Further, studies that had a theoretical framework for the intervention were found to be the most successful.

Web-based exercise programming is an emerging intervention approach to help meet the needs of college students during the COVID-19 pandemic. Aerobic and resistance training and mindful exercises have both shown promise in improving psychological health. Providing two popular forms of PA for college students may be beneficial for participation and engagement. However, studies examining the effectiveness and feasibility of these programs are scarce. To the best of our knowledge, few studies have compared the effects of PA (specifically aerobic exercise and resistance training) with mindful exercise on increasing PA and improving well-being concurrently in college students. Thus, the purpose of this study was to examine the feasibility and effectiveness of the Zoom-based aerobic and resistance exercise intervention (WeActive) and the Zoom-based mindful exercise intervention (WeMindful) in improving PWB, subjective vitality, and PA among college students over the course of eight weeks during an academic semester. We hypothesized that both groups would show an increase in PWB, and subjective vitality, and PA based on previous studies demonstrating that PA and yoga interventions are effective in increasing these outcomes. Further, we believed that the aerobic and resistance exercise group would experience a greater increase in moderate to vigorous PA compared to the mindful exercise group due to the participation in intervention activities which include moderate to vigorous exercise as part of the WeActive group lessons. This study will help researchers and interventionists further understand the benefits of different modes of exercise on improving PA and PWB through a web-based platform. This study uniquely contributes to the literature by comparing two popular forms of exercise and examining the effects on PA participation and PWB.

## 2. Methods

### 2.1. Participants and Study Design

In this study, participants were 77 college students enrolled in a large midwestern research university. There were 61 cisgender females, 6 cisgender males, and 5 transgender and gender nonconforming (TGNC) people. The average age was 23.43 (SD = 6), and approximately 65% of participants were white (see the full demographic breakdown in Table 1). Students were recruited via the University of Michigan's targeted email request system, the learning management system used by the university named Canvas, social media, and the undergraduate and graduate bulletins of the School of Kinesiology. Inclusion criteria for this study were (1) the ability to participate in intervention activities, (2) the ability to participate in all assessments, (3) completion of the consent form, and (4) enrollment as a student at the University of Michigan. Exclusion criteria were limited to the lack of ability to exercise regularly due to injury or illness. The study was approved by the University Institutional Review Board (IRB#HUM00189120/Ame00107415) of Health and Behavioral Sciences. Participants provided written consent prior to participation.

This study used a two-arm quasiexperimental design. All participants completed a baseline online Qualtrics survey within a week prior to random assignment into either WeActive or WeMindful intervention. Both interventions lasted 8 weeks during a winter/spring semester of 2021, and each participant completed a posttest online Qualtrics survey one week after the completion of the intervention. Both pre- and posttest Qualtrics surveys included measures on physical activity, psychological well-being, and subjective vitality. In addition, the baseline survey asked participants to report if they had regularly engaged in the following activities regularly over the last three months: (1) aerobic and strength exercises, (2) mindful exercises such as yoga, and (3) psychological and cognitive services. Participants were also asked to self-rate their overall health, on a five-point scale.

#### 2.1.1. Intervention Conditions and Components

*WeActive intervention group*. Participants attended two 30-minute Zoom-based exercise lessons per week for 8 weeks. Participants had the option to attend a live Zoom class or watch the recording of the session. The WeActive exercise lessons were taught by a student instructor who is a Certified Strength and Conditioning Specialist (CSCS) through the National Strength and Conditioning Association (NSCA) and had 5 years of experience teaching fitness classes and training. Each lesson consisted of a 5-minute warm-up with low-impact walking steps with dynamic stretching exercises, 20-minute aerobic and strength exercises, and a 5-minute cool-down with static stretching exercises. The strength training exercises typically included lower-body, upper-body, and core areas using circuit-training methods. The first four weeks focused mostly on strength, and the second four weeks involved more high-impact aerobic and strength training based on feedback from participants. When teaching each lesson, the instructor started with demonstrating each exercise and explained any modifications, then led the participants to perform the exercise with modification. The instructor provided learning cues and motivational cues throughout performing the exercises. At the end of each lesson, there was a quick review of the key points of the lesson. Each lesson video and the written lesson plan were posted on the Canvas study site to help participants repeat the lessons on their own time. Every week, we also sent an email notification to remind participants of their goals for the week and to provide positive reinforcement. The objective of the WeActive interventions was to help students engage in 150 minutes or more of moderate to vigorous PA per week and maintain the recommended amount of weekly PA throughout the course of eight weeks. We posted a variety of exercise videos to our study website, and we encouraged the participants to use the lessons multiple times over the week at their discretion and to participate with peers.

*WeMindful intervention group*. Participants attended two 30-minute Zoom-based mindful exercise lessons per week for 8 weeks. Participants had the option to attend a live Zoom class or watch the recording of the session. The WeMindful exercise lessons were taught by a senior student majoring in movement science. The student-instructor has had prior experience with group yoga for two years and has three years of experience teaching group exercise classes. Each lesson consisted of a 5-minute mindful warm-up focusing on mindful breathing, a 20-minute yoga practice, and a 5-minute mindfulness practice to close. The yoga practice typically included a flow of a variety of poses that increased with difficulty towards the latter weeks of the intervention. When teaching each lesson, the instructor demonstrated the yoga movement while providing learning cues, then led the participants in practicing the yoga pose and movement. The instructor used the tell-show-do methods to teach each of 4-6 yoga poses and transition movements. The instructor used similar teaching methods to engage the participants in performing all the poses and transition moves in a sequence. Throughout the process of leading the practices, the instructor provided learning cues and motivational cues to engage the participants in performing each yoga and transition move correctly. At the end of each lesson, the instructor guided the participants in a mindfulness practice by providing verbal cues. Each lesson video and the written lesson plan were posted on our study website to help participants repeat the lessons on their own time. Every week, we sent an email notification to remind participants of their goals for the week and to provide positive reinforcement.

### 2.2. Peer Coaching Component

In addition to the exercise conditions, we offered one 30-minute zoom-based peer coaching session per two weeks to help participants set goals, reflect on their strengths and priorities, self-monitor their progression towards their goals, and leverage their social support. These sessions were offered to all study participants. Participants were given journal prompts every two weeks to encourage self-awareness in relation to the topics listed above. Journal prompts were posted on the study website for participants to use. Additionally, we asked participants to share their exercise experiences, their thoughts and feelings about the exercise lessons, barriers or challenges they have faced, and any suggestions for the following weeks of the intervention. The strategies that were used in each session were grounded on the evidence-based behavior change techniques that have been effective in increase PA [49]. Each peer coaching session was also posted on the study website. The peer coaching sessions were run by a doctoral student who has been trained in motivational interviewing and an undergraduate student who majored in psychology.

### 2.3. Data Collection and Outcome Measures

The online questionnaire was distributed via Qualtrics one week prior to the start of the intervention and one week after the completion of the intervention. The questionnaire assessed PA, PWB, and subjective vitality. In addition, the baseline survey asked participants to report if they had regularly engaged in the following activities over the last three months: (1) aerobic and strength exercises, (2) mindful exercises such as yoga, and (3) psychological and cognitive services. Participants were also asked to self-rate their overall health, on a five-point scale. Further, intervention outcomes were also assessed during the posttest.

*Physical activity*. The International Physical Activity Questionnaire- (IPAQ-) Short Form is a 7-item measure to assess levels of PA intensity [50]. Participants were asked to recall the days, hours, and minutes spent on vigorous physical activity (VPA), moderate physical activity (MPA), and walking in the last week. PA scores were indicated by the metabolic equivalent (MET) minutes per week. VPA MET-minutes/week was calculated by multiplying the total minutes per week in VPA by 8.8. MPA MET-minutes/week was calculated by multiplying the number of minutes per week in MPA by 4.0. Walking MET-minutes/week was calculated by multiplying the number of minutes per week walking by 3.3. The total PA (TPA) MET-minutes/week was calculated by adding VPA MET-minutes/week, MPA MET-minutes/week, and walking MET-minutes/week. Examples of questions are “during the last 7 days, on how many days did you do moderate physical activities like carrying light loads, bicycling at a regular pace, or double tennis?” and “how much time did you usually spend doing moderate physical activities on one of those days?” This questionnaire has demonstrated internal reliability in both the baseline (Cronbach *α* = 0.72) and posttest (Cronbach *α* = 0.73) and is valid for use with young adults.

*Psychological well-being (PWB)*. The World Health Organization-Five Well-Being Index (WHO-5) is a short self-report assessment of current mental well-being [51]. The WHO-5 is comprised of the following five statements: (1) “I have felt cheerful and in good spirits,” (2) “I have felt calm and relaxed,” (3) “I have felt active and vigorous,” (4) “I woke up feeling fresh and rested,” and (5) “My daily life has been filled with things that interest me.” Participants were asked to indicate which response that felt closest to how they have felt over the last two weeks on a 6-point rating scale, ranging from 5 = all the time to 0 = at no time. Total scores are calculated by adding the five items. The WHO-5 had high internal reliability in this study at both baseline (Cronbach *α* = 0.85) and posttest (Cronbach *α* = 0.89).

*Vitality*. The Subjective Vitality Scale (SVS) was designed to assess individuals' feelings of vitality using 7 items on a 7-point rating scale [11]. Vitality is highly related to eudemonic well-being. The participants were asked to indicate the degree to which the statements are true for their lives. Responses range from 1 = not true at all to 7 = very true. Sample items include “I feel alive and vital,” “I look forward to each new day,” and “I feel energized.” The total score was calculated by averaging the scores of the individual items. The SVS had high internal reliability in this study in both the baseline (Cronbach *α* = 0.9) and posttest (Cronbach *α* = 0.89) scores.

*Intervention feasibility and acceptability*. The Assessing Intervention Feasibility and Acceptability (AIFA) questionnaire was designed to measure intervention implementation outcomes [52]. Participants completed the 12-item AIFA on a 5-point rating scale at the conclusion of the intervention. The AIFA consisted of three subscales: acceptability, appropriateness, and feasibility. Each subscale had 4 items. Sample items include the following: “The intervention strategies are appealing to me” (acceptability), “The intervention components seem applicable” (appropriateness), and “The intervention components seem doable” (feasibility).

Participants were asked to rate their agreement with each statement on a 5-point rating scale, ranging from 5 = strongly agree to 1 = strongly disagree. Scores for each subscale were calculated by adding up the items that correspond to the subscale. Total scores were calculated by adding all the items on the survey. This questionnaire demonstrated internal reliability (Cronbach *α* = 0.94) in this study.

### 2.4. Data Analysis

Among 77 participating students, three were excluded due to lack of participation and five were excluded from the final data analysis due to missing data in the outcome measures. The missing data were screened by using listwise deletion. The final data set included 71 participants, 43 in the WeActive intervention group and 28 in the WeMindful intervention group.

An independent sample *t*-test was performed on the baseline data to determine if there were significant differences in the outcome variables and the demographic variables between the two groups in order to determine which type of analysis was needed to examine the data. The results showed an overall significant difference in mean scores of the baseline PWB scores between the two groups, *t*(66.59) = −1.96, *p* = 0.054. Additionally, there was a significant difference in participants' reports of attending psychological or cognitive services in the last three months by group, *t*(49.15) = 2.53, *p* = 0.015. Since significant differences were observed at baseline, a repeated-measures analysis of covariance (ANCOVA) was used to examine the effects of the intervention on posttest PA variables, PWB, and vitality by group while statistically controlling for covariates (pretest PWB scores and psychological service attendance). The between-factor was the intervention group (WeActive vs. WeMindful), and the within-subjects factor was time (baseline vs. posttest).

## 3. Results

Table 2 present the baseline and posttest scores by group. Skewness and kurtosis were checked for normality, and logistic transformations were performed on PA data due to violations of normality. Regarding the PA outcome variables, the IPAQ scoring guidelines indicate that individuals who engage in at least 1500 MET-minutes/week of VPA or at least 3000 MET-minutes of TPA are considered “high” active. Individuals who engaged in less than 3000 MET-minutes/week of TPA but greater than 600 MET-minutes/week of TPA were considered “moderate” active [53]. Individuals who engaged in below 600 MET-minutes/week of TPA were considered “low” active.  Table 3 displays the percentage by group of participants who are considered “high,” “moderate,” and “low” active both by baseline and posttest. Both groups observed an increase in the number of participants who were considered “high” and “moderate” active.

Regarding PWB well-being scores, scores below 13 indicate poor well-being. Both groups in this study were below 13 for both the pretest and posttest [54]. However, the WeActive group saw a decrease in PWB scores from baseline to posttest while WeMindful saw an increase in PWB scores from baseline to posttest. Regarding subjective vitality, both groups saw an increase in vitality scores from baseline to posttest.

### 3.1. Intervention Effects on Physical Activity

Table 4 shows the results of a repeated-measures ANCOVA for all PA variables. While controlling for pretest PWB and psychological services attended, repeated-measures ANCOVA showed that there was no significant main effect of group and no significant interaction of time with group for weekly walking, VPA, MPA, and TPA MET-minutes. However, there was a significant main effect of time for TPA (*F* = 7.89, *p* = 0.006, *η*^2^ = 0.049), VPA (*F* = 5.36, *p* = 0.024, *η*^2^ = 0.022), and walking (*F* = 7.34, *p* = 0.009, *η*^2^ = 0.042). There was no main effect of time for MPA. The results indicate that both groups experienced an increase in TPA, VPA, and walking after the 8-week interventions.

### 3.2. Intervention Effects on Psychological Well-Being and Subjective Vitality

Table 5 shows the results of a repeated-measures ANCOVA for PWB and subjective vitality. While controlling for psychological services attended, repeated-measures ANCOVA showed that there was no significant main effect of group and of time for PWB. However, there was a significant interaction between time and group for PWB (*F* = 11.26, *p* = 0.001, *η*^2^ = 0.022).

**Figure 1:**
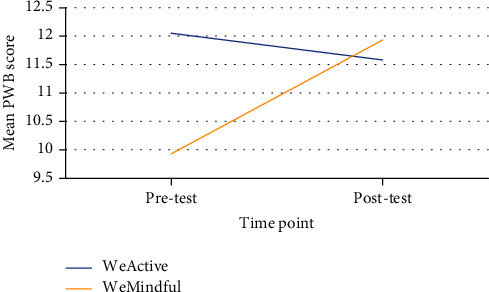
Interaction effect for psychological well-being (PWB) scores from pretest to posttest by group.

Figure 1 displays the interaction effect for PWB scores over time by group. The results indicated that participants in the WeActive group experienced a decrease in PWB scores while participants in the WeMindful group experienced an increase in PWB scores over time.

**Figure 2:**
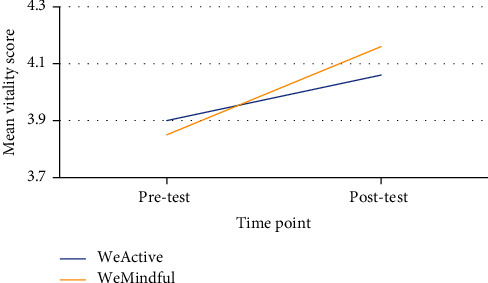
Main effect of group for subjective vitality from pretest to posttest.

While controlling for pretest PWB and psychological services attended, a repeated-measures ANCOVA revealed no significant main effect of time and no significant interaction effect of group and time for subjective vitality. However, there was a significant main effect of group for subjective vitality (*F* = 8.91, *p* = 0.007, *η*^2^ = 0.088).  Figure 2 displays the main effect of group for subjective vitality. These results indicated that the WeMindful group experienced an overall greater increase in subjective vitality than the WeActive group.

### 3.3. Feasibility and Acceptability of the Intervention

Table 6 displays the mean score by group on the intervention feasibility and acceptability scale. As seen in Table 6, overall, the participants found the intervention to be acceptable, appropriate, and feasible. There were no differences by group in the rating of the feasibility and acceptability of the intervention implementation. Scores for each subscale range from 4 to 20, and the total scale ranges from 12 to 60. Both the WeActive and WeMindful groups rated the feasibility the highest of the three subscales. The total score for both groups was approximately 50 out of 60.

## 4. Discussion

The purpose of this study was to investigate the feasibility and effectiveness of the web-based WeActive and WeMindful interventions in improving physical activity (PA), psychological well-being (PWB), and subjective vitality among college students. Aerobic and resistance training and mindful exercise are popular forms of exercises that have been previously shown to be effective in improving PA and PWB. Moreover, web-based interventions may have helped meet the needs of students during the COVID-19 pandemic.

Both the WeActive and WeMindful groups demonstrated an increase in TPA, VPA, and walking over time. The WeActive group experienced a decrease in PWB scores while the WeMindful group experienced an increase in PWB scores over time. The WeMindful group experienced a greater increase in subjective vitality than the WeActive group. Further, the participants found the intervention to be acceptable, appropriate, and feasible. Although the effect sizes were small, even modest increases in PA, PWB, and subjective vitality have shown to provide health benefits [55–57].

As hypothesized, the WeActive group experienced an increase in TPA, VPA, and walking after the 8-week intervention. However, we did not expect an increase in PA for the WeMindful group; specifically, we did not expect an increase in VPA. WeMindful sessions were intended to be low-intensity exercises that were focused on flexibility and balance. Therefore, any increases in VPA would have likely taken place outside of the intervention. It is possible that through participation in mindfulness practices and yoga, students became more aware of their physical bodies and fitness and were more motivated to participate in PA in general, explaining the increase in PA experienced by WeMindful participants from baseline to posttest. In addition, since this intervention took place during the COVID-19 pandemic, there may have been restrictions on gyms and exercise activities at the start of the intervention that may have been lifted towards the end of the intervention, allowing for greater exercise participation in general. Further, previous studies using behavior change techniques such as self-monitoring and self-regulation have been shown to be effective in promoting and maintaining PA [49]. Our study utilized these methods in the peer coaching sessions, such as goal-setting and self-reflection, each week to help achieve meaningful change over the course of the 8-week intervention.

The mindful exercise intervention in this study primarily consisted of yoga-based practices with short mindfulness meditations at the beginning and end of each session. Previous studies have found that college students randomly assigned to either a yoga-only or mindfulness 8-week intervention experienced similar benefits in terms of improvement in depressive, anxiety, and stress symptoms from baseline to follow-up in both conditions [58]. Our findings of an increase in PWB after a mindful exercise intervention are consistent with previous research. A study of 30 students aged 18 to 22 found a significant increase in PWB after a yoga intervention that consisted of a one-hour practice for 30 sessions [59]. Medical students who participated in a 6-week yoga and meditation intervention found significant improvement in hedonic aspects of well-being, such as happiness, positivity, and satisfaction [60]. However, our findings that the WeActive participants experienced a decrease in PWB are inconsistent with other findings. PA has been found to be positively related to PWB in college students [16, 61]. Research by Herbert and colleagues found that 6-week low to moderate aerobic exercise intervention conducted with college studies found significant improvements in self-reported mental health, even during stressful periods such as exams [38].

However, there are studies that show a difference in PWB or related mental health constructs when comparing participant outcomes after engaging in either a mindful exercise condition or aerobic exercise condition. A study on adults aged 18-49, who participated first in a one-hour yoga session, then completed a one-hour aerobic exercise session a week later, found that participants experienced a significant decrease in anxiety after the yoga class, but not after the aerobic exercise session [62]. Research has shown that older adults who regularly participated in either yoga or Tai Chi at least one 60-minute session per week experienced better mood and mental health when compared with older adults who regularly participated in a 60-minute aerobic exercise class [63]. Further, it has been previously reported that college students who participated in yoga experienced lower levels of anxiety, depression, tension, anger, and confusion than participants in swimming, body conditioning, fencing, or lecture-control conditions [64]. Still, some other studies have reported no differences in PWB and related constructs between mindful exercise and aerobic or resistance training activities. A study of the effects of a 90-minute mindful exercise session compared to a 90-minute aerobic exercise session on mood found that both mindful exercise and aerobic activities enhanced mood and subjective well-being after a single session among 322 females [65]. Conversely, another study of college students enrolled in a 4-week program where students attended seven 30-minute exercise classes that consisted of activities such as yoga and dancing found no significant increase in PWB [66]. Due to these inconsistencies in the research, further exploration of the relationship between PWB and different types of exercise conditions is warranted.

Our study found a greater improvement in subjective vitality in the WeMindful group than in the WeActive group. This is supported by other research that has found an increase in vitality after mindful exercise interventions. A randomized control trial on 109 graduate students found that participants who received a 25-minute yoga-based practice twice a week over seven weeks had greater increases in their subjective vitality scores when compared to a control group [67]. A study on 30 college students who participated in mindful exercise (Tai Chi) twice per week for 3 months found an increase in vitality and mental health when compared to their baseline scores [68]. Mindful exercises, such as yoga, appear to positively influence subjective vitality.

The strengths of this study are that both Zoom-based interventions were appropriate, acceptable, and feasible for college students to participate, with an average overall rating of approximately 50 out of 60 possible points. Understanding participants' perception of the Zoom-based intervention is essential to recognizing where implementation may have succeeded or failed and determining the fit of the intervention to the current context. An additional strength of this study was flexible options in terms of participation. Weekly lessons were offered live for participants to attend. Lessons were also recorded and immediately uploaded to the study site where participants could access the videos at any time if they were unable to attend the live session. The study team also sent reminders to complete the exercise sessions, which has been shown to improve effectiveness in virtual interventions [47]. Web-based interventions have the ability to reach a wider population due to increased access to the internet. At the time of this study, data show that 93% of American adults are able to use the internet [69]. Further, there is a relatively low cost to implementing web-based interventions and there is greater convenience to materials due to the unlimited access to the study website. Virtual interventions may prove to be an effective and innovative avenue of promoting positive health behaviors.

There are several limitations to note as well. This study is limited to examining the two web-based exercise intervention effects on PA and PWB. However, to better understand the beneficial effects of these exercises on PA and PWB, including a true control group in future studies may be warranted. Further, given the fact that the participants have the flexibility for attending either live or recorded lessons, it was also difficult to determine the fidelity of participants to the study sessions. Attrition is often a concern with internet-based interventions [45]. Therefore, further studies should employ thorough fidelity and attrition measures to inform adherence to study protocol. In addition, the intervention took place during the COVID-19 pandemic which has provided unprecedented challenges worldwide. Changes to participants' self-report scores may have been unduly influenced by the current state of the world. Further limitations include unequal sample sizes and lack of gender or race diversity in the participant groups. Approximately 63% of our sample identified as white, and 65% of our sample identified as cisgender females. Future studies should focus on using objective measures of study variables as well as recruiting a more diverse sample. Research has shown that females engage in less PA than males and historically there has been a paucity of research on women's PA [70, 71]. Although there may be a gender bias in this study due to the large number of female participants, the results of this study may contribute to the literature by informing how to increase PA in females.

Despite limitations, it is important to note that there are several practical implications from this study. First, this study adds to the growing literature on online interventions and their effectiveness in improving PA and PWB. Secondly, there are likely to be lasting mental and physical health effects due to the pandemic. Results from this study demonstrated that a virtual program can help students increase their PA and that mindful exercise might be particularly effective in improving PWB. Universities should consider providing exercise programs to help promote both the physical and psychological wellness of their students. Finally, the COVID-19 pandemic has revealed the extensive possibilities of virtual learning and connecting. Evidence from this study may provide support for virtual programming that may increase overall accessibility to interventions and overcome some of the limitations found in traditional in-person programs.

## 5. Conclusion

To conclude, the WeActive and WeMindful interventions were effective in increasing PA among participants in both groups. Further, the WeMindful group experienced an increase in PWB while the WeActive group reported a decrease in PWB over time. Finally, the WeMindful group experienced a greater increase in subjective vitality when compared to the WeActive group. Participants found the intervention to be acceptable, appropriate, and feasible. This study demonstrated that mindful exercise is effective in increasing PA, PWB, and subjective vitality while aerobic and strength training may only be effective in increasing PA. Further research is warranted to explore the different effects of aerobic and strength training when compared to mindful exercises on well-being and subjective vitality.

## Figures and Tables

**Table 1:** Demographic data of total participant group.

Variables	*n*	%
Gender		
Cisgender female	60	65%
Cisgender male	6	31%
Transgender & gender nonconforming (TNGC)	5	4%
Race		
Asian	14	20%
Black or African American	4	6%
White	45	63%
Multiracial	8	11%
Ethnicity		
Hispanic	6	8%
Non-Hispanic	65	90%
Education status		
1^st^ year	7	10%
2^nd^ year	9	13%
3^rd^ year	15	21%
4^th^ year+	14	19%
Master's	12	17%
Professional	2	3%
Doctoral	12	17%

**Table 2:** Descriptive statistics by group at pretest and posttest.

Variable	*n*	Pretest		Posttest	
		Mean	SD	Mean	SD
Total PA (MET-min/week)					
WeActive	43	1283.65	1179.04	1770.48	1546.43
WeMindful	28	1150.02	1052.89	1553.73	901.388
Vigorous PA (MET-min/week)					
WeActive	43	559.07	760.53	736.74	883.078
WeMindful	28	341.14	577.61	568.57	700.675
Moderate PA (MET-min/week)					
WeActive	43	195.81	296.06	390.23	766.332
WeMindful	28	214.29	302.48	187.86	175.317
Walking (MET-min/week)					
WeActive	43	528.77	501.43	643.50	594.033
WeMindful	28	594.59	914.03	797.30	634.689
Psychological well-being					
WeActive	43	12.05	5.10	11.58	4.851
WeMindful	28	9.93	3.99	11.93	5.497
Vitality					
WeActive	43	3.90	1.40	4.06	1.369
WeMindful	28	3.85	1.12	4.16	1.428

**Table 3:** Percentage of participants by group who are “high,” “moderate,” and “low” active at baseline and posttest.

PA level	Group	Pre	Post
High	WeActive	12%	19%
	WeMindful	7%	11%
Moderate	WeActive	56%	60%
	WeMindful	61%	79%
Low	WeActive	33%	21%
	WeMindful	32%	11%

**Table 4:** Results of a repeated-measures ANOVA for all PA variables.

Effects	Total			Vigorous			Moderate			Walking		
	*F*	*p*	*η* ^2^	*F*	*p*	*η* ^2^	*F*	*p*	*η* ^2^	*F*	*p*	*η* ^2^
Group	0.6	0.443	0.005	0.21	0.647	0.002	2.02	0.16	0.018	2.09	0.152	0.018
Therapist	0.56	0.456	0.005	1.82	0.182	0.019	2.15	0.147	0.019	1.01	0.318	0.009
Pre_PWB	4.16	0.045	0.034	4.01	0.049	0.041	1.96	0.166	0.018	1.09	0.3	0.01
Time	7.89	0.006	0.049	5.36	0.024	0.022	2.22	0.14	0.013	7.34	0.009	0.042
Time∗group	0.41	0.526	0.003	0.07	0.787	<0.001	0.14	0.711	<0.001	0.01	0.931	<0.001
Time∗therapist	2.53	0.116	0.016	0.07	0.794	<0.001	0.22	0.639	0.001	3.16	0.08	0.019
Time∗pre_PWB	0.02	0.882	<0.001	0.07	0.789	<0.001	0.04	0.841	<0.001	0.08	0.773	<0.001

**Table 5:** Results of a repeated-measures ANOVA for psychological well-being (PWB) and vitality.

Effects	PWB			Vitality		
	*F*	*p*	*η* ^2^	*F*	*p*	*η* ^2^
Group	0.02	0.89	<0.001	8.91	0.007	0.088
Therapist	4.46	0.038	0.054	3.1	0.083	0.032
Pre_PWB	—	—	—	120.75	0.001	0.565
Time	1.13	0.292	0.002	1.99	0.163	0.008
Time∗group	11.26	0.001	0.022	0.9	0.345	0.004
Time∗therapist	4.34	0.041	0.009	1.09	0.083	0.013
Time∗pre_PWB	—	—	—	1.42	0.238	0.006

**Table 6:** Means and SD of intervention feasibility by total score and subscale by group.

	Acceptability		Appropriateness		Feasibility		Total	
	Mean	SD	Mean	SD	Mean	SD	Mean	SD
WeActive	15.85	2.60	15.95	2.60	17.47	2.28	50.12	6.40
WeMindful	15.93	2.34	15.93	2.34	17.36	1.93	49.64	6.38

## Data Availability

The data that support the findings of this study are available on request from the corresponding author. The data are not publicly available due to restrictions, e.g., they are containing information that could compromise the privacy of research participants.
